# Ultra-broad hybrid capture-based targeted next-generation sequencing for sensitive plasma pathogen cfDNA detection in bloodstream infections

**DOI:** 10.1186/s12967-025-07258-9

**Published:** 2025-10-31

**Authors:** Muyun Wei, Xiangzhao Ai, Dejian Gu, Shengyang Zhang, Ke Xu, Shuangshuang Li, Shaowei Mao, Min Li

**Affiliations:** 1 Department of Laboratory Medicine, Ren Ji Hospital, Shanghai Jiao Tong University School of Medicine, No.160 Pujian Road, Shanghai, China; 2 Shanghai Jiao Tong University School of Nursing, Shanghai, China; 3 Jiading Branch, Ren Ji Hospital, Shanghai Jiao Tong University School of Medicine, Shanghai, China; 4 Department of Bioengineering, School of Life Sciences and Biotechnology, Shanghai Jiao Tong University, No. 600 Dongchuan Road, Shanghai, China; 5 Geneplus-Beijing Co., Ltd., Beijing, China

**Keywords:** Ultra-broad hybrid capture-based targeted next-generation sequencing (tNGS), CfDNA, Bloodstream infections (BSI), Immunocompromised patients, Metagenomic next-generation sequencing (mNGS)

## Abstract

**Background:**

The limited genomic targeting range of current targeted next-generation sequencing (tNGS) workflows results in limited detection of pathogen-derived cell-free DNA (cfDNA), making it challenging to apply this approach to bloodstream infections (BSIs). Here, we developed an ultra-broad hybrid capture-based tNGS method to detect plasma pathogen-derived cfDNA and evaluate its suitability for the diagnosis of BSI.

**Methods:**

This study introduced an ultra-broad hybrid capture-based tNGS method featuring an ultra-broad pathogen panel (1872 pathogens) and high-density probe coverage. To adequately evaluate its performance, we conducted retrospective tests in 208 suspected BSI patients (139 immunocompromised), comparing tNGS results with mNGS, conventional microbiological testing (CMT), and comprehensive clinical diagnoses.

**Results:**

In pathogen detection, the concordance between ultra-broad hybrid capture-based tNGS and mNGS results was 93.75%. The diagnostic accuracy of tNGS in BSI was comparable to mNGS (76.44% vs. 75.00%) and significantly higher than CMT (45.67%, *p* < 0.0001). In immunocompromised populations, the diagnostic accuracy of tNGS was similar to mNGS (77.70% vs. 76.98%). tNGS detected 92.09% (163/177) of pathogens identified by mNGS. Two of the missed pathogens were not included in the 1872 pathogens panel, and both were from the immunocompromised group.

**Conclusions:**

Ultra-broad hybrid capture-based tNGS exhibits sensitivity and accuracy comparable to mNGS, effectively covering a relatively wide range of pathogens, and may serve as an economic screening tool for BSI in the future.

**Supplementary Information:**

The online version contains supplementary material available at 10.1186/s12967-025-07258-9.

## Background

The mortality rate of bloodstream infections (BSI) exceeds 30%, ranking first among infectious diseases [1]. Particularly for immunocompromised patients, the incidence of BSI reaches 28%, leading to significant morbidity and clinical burden [2]. Studies on BSI patients have shown that timely and accurate antimicrobial therapy is crucial for improving patient prognosis [1, 3, 4]. Blood culture remains the gold standard for diagnosing BSI [5]. However, its long turnaround time and low detection rate fail to meet the clinical demand for both timeliness and sensitivity in BSI diagnosis [5, 6]. Although polymerase chain reaction (PCR) methods, independent of microbial culture, have improved the detection of pathogens associated with BSI, their limited coverage of target pathogens still falls short of meeting the diagnostic needs for difficult-to-culture, rare, or unusual pathogens, as well as polymicrobial infections, particularly in immunocompromised patients [7, 8]. Metagenomic next-generation sequencing (mNGS) enables unbiased detection of all nucleic acids in a sample, identifying the presence of bacteria, viruses, fungi, and parasites, and significantly improving infection surveillance across diverse sample types, including BSI. Numerous retrospective studies have confirmed the enhanced clinical performance of mNGS for BSI diagnosis [9–11]. However, the high sequencing data requirements of mNGS limit its widespread adoption due to cost constraints.

Approaches such as multiplex PCR-based targeted amplification or probe-based enrichment can effectively amplify pathogen nucleic acid signals, reducing sequencing data requirements and significantly lowering costs (one-third to half the price of mNGS) [10, 12–15]. Currently, multiplex PCR-based tNGS workflows are widely used in the diagnosis of lower respiratory tract infections (LRTIs), with their cost-effectiveness and sensitivity being clinically validated. However, there is a growing consensus that their poor detection performance for blood cell-free DNA (cfDNA) renders them currently unsuitable for diagnosing BSI. Additionally, the limited pathogen target panels of multiplex PCR restrict their applicability in immunocompromised populations.

Here, we introduced an ultra-broad hybrid capture-based tNGS method to address these limitations. This method employs a high-density probe tiling strategy to enhance coverage of cfDNA fragments, thereby improving detection sensitivity for bloodstream cfDNA. It features an ultra-broad pathogen panel, including over 1,800 species, to cover the spectrum of pathogens affecting the immunocompromised population. We conducted a retrospective comparative analysis involving 208 suspected BSI patients (including 139 immunocompromised individuals) to evaluate the diagnostic performance of the ultra-broad hybrid capture-based tNGS against mNGS and conventional microbiological testing (CMT).

## Methods

### Study design and sample collection

This study collected 361 plasma samples (residual from mNGS testing) from hospitalized patients with suspected BSI at Renji Hospital, Shanghai Jiao Tong University School of Medicine, between April 2022 and December 2023. Based on definitive CMT results and complete clinical information, a total of 208 patients were enrolled, including 139 immunocompromised patients and 69 immunocompetent patients. A total of 153 patients were excluded due to insufficient remaining blood samples for tNGS testing (*n* = 83), the absence of available paired blood culture results (*n* = 32), or incomplete medical histories (*n* = 38). This study was approved by the Ethics Committee of Renji Hospital (Approval No. KY2023-109-C). All patients provided written informed consent to donate residual samples for scientific research.

CMT results included a variety of blood tests, such as blood cultures (BC) within seven days, PCR tests for viruses, and results of the G and GM tests on blood. Blood samples (both aerobic and anaerobic bottles) were incubated in automated BC systems for a maximum of 5 days (BD BACTEC FX; BD Biosciences). The pathogens in the positive cultures were identified using matrix-assisted laser desorption/ionization-time of flight mass spectrometry (bioMérieux, Marcy-l’Étoile, France).

Immunocompromised status is defined by the presence of one or more of the following risk factors [16]: (A) hematologic cancer; (B) chemotherapy within the last three months; (C) chronic steroid (> 0.3 mg/kg/day of prednisone-equivalent for ≥ 3 weeks) or biologic drug use for autoimmune diseases or other immunosuppressive therapies; (D) solid organ transplant within the last six months; (E) neutropenia; (F) acquired or inherited severe immunodeficiency.

### Definitions and diagnosis of bloodstream infection

To enable a more accurate and comprehensive assessment of the diagnostic performance, we employed the diagnostic criteria for BSI proposed in previous studies [9, 17], classifying patients into definite BSI, probable BSI, possible BSI, or unlikely BSI (Supplementary Table 1). This allows for the identification of a broader range of pathogens associated with clinical symptoms of BSI and the detection of BSI that blood culture (BC) cannot diagnose. Two experienced ICU physicians made the final clinical determination for each patient, considering clinical data, including clinical manifestations, complications, underlying diseases, Sequential Organ Failure Assessment (SOFA) scores, treatment interventions, clinical outcomes, and all microbiological tests collected from potential infection sites within seven days before and after enrollment (including tests from blood and local lesions samples; as well as blood mNGS and CMT results).

### The workflow of ultra-broad hybrid capture-based tNGS

The detailed process is shown in the Supplementary Methods. In brief, cfDNA was extracted from plasma using a cfDNA extraction kit (Geneplus, Suzhou, China), followed by the construction of a pre-library using a library construction kit (Yeasen, Shanghai, China). Subsequently, the library was incubated with Geneplus probes (covering 1872 microbes) for 4 h to complete the library construction (Gene + GIN96 [Geneplus, Suzhou, China]; Supplementary Table 2). Sequencing was performed on the Gene + Seq-100 platform with a sequencing depth of 5 M reads. The generated data was processed through an in-house developed automated analysis solution. Finally, based on a total of 1 million sample reads, reads consistent with individual organisms were normalized to generate reads per million (RPM) metrics. Microbes with RPM exceeding a specified threshold were selected for further analysis and interpretation of results. The threshold was set at RPM ≥ 6 for common pathogens (excluding mycobacteria) and ≥ 0.5 for fungi and mycobacteria, as in previous studies [14, 18, 19].

### The workflow of mNGS

mNGS sequencing was performed on the BGI platform. The detection process is as previously reported [20–22]. In brief, cell-free DNA (cfDNA) was directly extracted from plasma and prepared through end-repair, adapter ligation, and amplification. Sequencing was conducted using the MGISEQ-2000 platform with a single-end 50 bp strategy (20–40 million reads for each sample). Reads that were short (< 35 base pairs), of low quality, and human sequences were filtered out, and the remaining reads were aligned against an internal database for the identification of microbial species. The reporting of pathogenic microorganisms mainly followed the results of previous studies [22].

### Statistical methods

The primary outcome was to assess the diagnostic performance of tNGS based on the clinical diagnosis of BSI and compare it with that of CMT and mNGS. The comparison of NGS with clinical testing referred to the Karius method [9]. Continuous variables are expressed as medians and interquartile ranges (IQR), while categorical variables are expressed as frequencies and percentages. Group comparisons were performed using the unpaired t-test and Mann-Whitney U test. For comparisons between groups of categorical variables, the chi-square test was used. Statistical comparisons were conducted using SPSS 26.0 (SPSS Inc., Chicago, Illinois, USA). All graphics were created using GraphPad Prism (version 9.2.0) and R software (version 4.3.1; http://www.r-project.org, The R Foundation). P-values less than 0.05 were considered statistically significant. The significance threshold level for correlation coefficients was adjusted for multiple comparisons using the Bonferroni correction.

## Results

### Patient diagnostic category and microbiologic results

The study finally included 208 patients. The median age of the patients was 59 years, and 53.85% (112/208) were male. Among 139 immunocompromised patients, ninety-five patients (45.67%) were on long-term corticosteroid therapy due to solid organ transplantation or autoimmune diseases, and 36 patients (17.31%) were undergoing chemotherapy for solid tumors or hematological malignancies. Demographic characteristics and baseline features are detailed in Table 1. There were no significant differences in clinical characteristics between immunocompromised and immunocompetent patients.

**Table 1:** Demographic and clinical characteristics of patients included in this study

Characteristic	Total	Immuno- compromised	Immuno- competent	*P*-value
**Number**,** n (%)**	208(100%)	139(66.83%)	69(33.27%)	
**Male gender**,** n (%)**	112(53.85%)	74(53.24%)	38(55.07%)	0.24
**Age (year)**,** median (IQR)**	59(44, 69)	58(44, 68)	59(47, 69)	0.63
**Comorbidities**, **n (%)**				0.66
Diabetes	37(17.79%)	22(15.83%)	15(21.74%)	
Cardiovascular/cerebrovascular diseases	26(12.50%)	16(11.51%)	10(14.49%)	
Surgery(within 14 days)	47(22.60%)	37(26.62%)	10(14.49%)	
**Immunocompromised status**,** n (%)**				
Hematologic malignancy	19(9.13%)	19(13.67%)	NA	
Solid-organ transplantation	64(30.77%)	64(46.04%)	NA	
Receiving chemotherapy	17(8.17%)	17(12.23%)	NA	
Prolonged corticosteroid therapy ^a^	8(3.85%)	8(5.76%)	NA	
Autoimmune disease	31(14.90%)	31(22.30%)	NA	
**Antibiotics therapy**				0.079
No prior antibiotics	55(26.44%)	31(22.30%)	24(34.78%)	
Prior antibiotics	153(73.56%)	108(77.70%)	45(65.22%)	
**Clinical manifestations**,** n (%)**				
Respiratory	85(40.87%)	61(43.88%)	24(34.78%)	
Gastrointestinal	12(5.77%)	9(6.47%)	3(4.35%)	
Urinary	27(12.98%)	23(16.55%)	4(5.80%)	
Sepsis	65(31.25%)	36(25.90%)	29(42.03%)	
Cardiovascular	16(7.69%)	9(6.47%)	7(10.14%)	
Neurological	3(1.44%)	1(0.72%)	2(2.90%)	
**Laboratory findings**				
WBC (10^9^/L), median (IQR)	7.81 (4.62, 11.57)	6.44 (3.88,11.14)	8.92 (6.13, 13.56)	0.82
Lymphocyte ratio (%), median (IQR)	18.25 (14.63, 24.03)	17.15 (14.63, 13.33)	20.35 (15.42,27.77)	0.71
SOFA score, median (IQR)	6 (3, 9)	6 (3, 9)	6 (3, 8)	0.93
**Outcome**,** n (%)**				0.013
Survival	175(84.13%)	124(89.21%)	51(73.91%)	
Death	24(11.54%)	11(7.91%)	13(18.84%)	
Unknown	9(4.33%)	4(2.88%)	5(7.25%)	

Abbreviations: IQR, interquartile range; WBC, White blood cell; NA, not available

^a^ Defined as > 0.3 mg/kg/d of prednisone-equivalent for ≥ 3 weeks

*P*-values < 0.05 were considered statistically significant

**Fig. 1:**
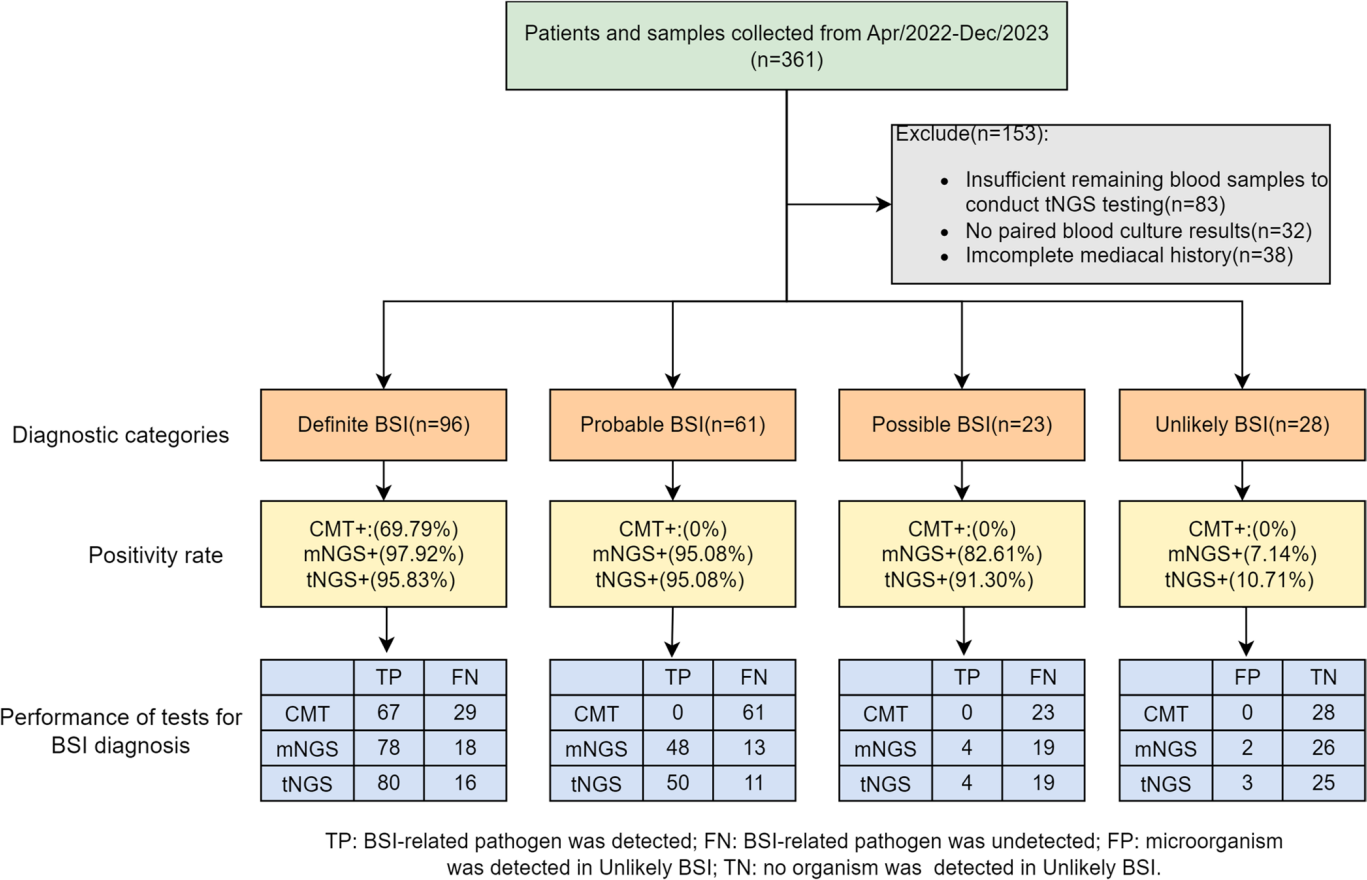
Patient enrollment and three methods result in different diagnostic categories. Diagnostic categorization was completed according to the rules described in previous studies (Methods section). Positivity rate: refers to tests with organisms detected/total tests of tNGS, mNGS, and CMT within each diagnostic category. +: Positive, -: Negative. Performance of tests for BSI diagnosis: refers to whether tNGS, mNGS, and CMT can detect pathogens related to BSI symptoms in patients of each diagnostic category, with results classified as true positive (TP), false positive (FP), true negative (TN), and false negative (FN)

After diagnostic classification, the proportion of definite BSI was 46.15% (96/208), while the proportions of probable BSI, possible BSI, and unlikely BSI were 29.33% (61/208), 11.06% (23/208), and 13.46% (28/208), respectively. Among the 208 blood samples, the positive rates (at least one microorganism detected) of mNGS, tNGS, CMT, and blood culture were 83.17% (173/208), 83.65% (174/208), 32.21% (67/208), and 22.12% (46/208), respectively. The proportion of each BSI subgroup and the detection rates of these methodologies are presented in Fig. 1.

### The performance of pathogen detection of ultra-broad hybrid capture-based tNGS, compared with mNGS and CMT

**Fig. 2:**
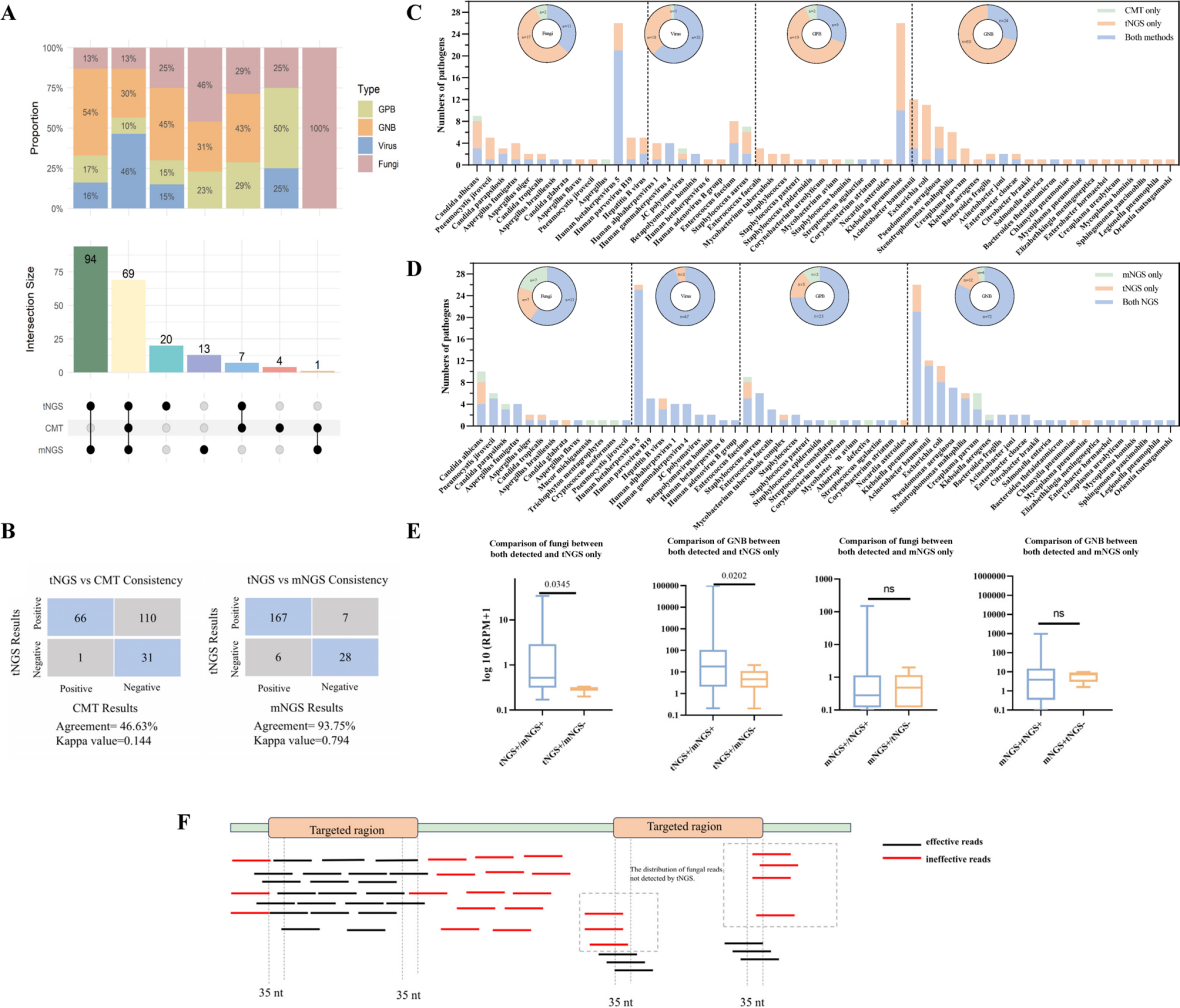
Detection performance of pathogens between tNGS, mNGS, and CMT. (**A**) Count and intersection size of pathogens detected by three methods. GPB: Gram-positive bacteria; GNB: Gram-negative bacteria. (**B**) Consistency in detection results using tNGS, mNGS, and CMT. tNGS vs. reference method, Positive-Positive: tNGS identified at least one microorganism consistent with the reference method (CMT/mNGS); Positive-Negative: tNGS additionally detected microorganisms while the reference method was negative. Negative-Positive: tNGS failed to detect any microorganisms found by the reference method; Negative-Negative: Neither tNGS nor the reference method detected microorganisms. (**C**) The bar chart illustrates the numbers of CMT and tNGS in detecting each pathogen. The sunburst chart summarizes the total number of detections for each taxon (bacteria, fungi, and viruses) by both methods. The numbers in the chart represent the count of detections. (**D**) Comparison of pathogen detection between tNGS and mNGS. The description of the details in the figure can refer to C. (**E**) Comparison of RPM between tNGS+/mNGS- and tNGS+/mNGS + groups of fungi and GNB. Comparison of RPM between mNGS+/tNGS + and mNGS+/tNGS- groups of fungi and GNB. (**F**) A schematic representation of the distribution of fungal reads not detected by tNGS. Nt: nucleotide

A total of 121 bacteria, 36 fungi, and 51 viruses were identified in the blood using three different methods. tNGS identified 190 pathogens in 64.42% (134/208) of the samples, while mNGS identified 177 pathogens in 62.50% (130/208) of the samples. The positive rate of tNGS was comparable to that of mNGS (*p =* 0.64) and was significantly higher than that of CMT (32.21%, *p* < 0.0001). Among these pathogens, 69 pathogens were commonly detected by all three methods. The most common pathogens were *Klebsiella pneumoniae* (*n* = 26), Human herpesvirus 5 (*n* = 26, also known as Human cytomegalovirus), Acinetobacter baumannii (*n* = 12), Escherichia coli (*n* = 11), and Candida albicans (*n* = 10). Compared to the other two methods, tNGS detected 20 additional pathogens (12 bacteria, 5 fungi, and 3 viruses). However, 13 pathogens detected by mNGS were also missed by the tNGS test, with 46% being fungi (Fig. 2A). We assessed the concordance of tNGS with CMT and mNGS in detecting results from blood samples, that is, whether they could provide identical detection results. The detection concordance between tNGS and mNGS was high at 93.75%, with a kappa value of 0.794, whereas the concordance with CMT was only 46.63% (Fig. 2B).

When comparing the consistency of pathogen detection between tNGS and CMT, tNGS detected more pathogens (91.35% vs. 38.94%, *p* < 0.0001), regardless of whether they were bacteria (92.56% vs. 28.93%, *p* < 0.0001), fungi (77.78% vs. 36.11%, *p* < 0.0001), or viruses (98.04% vs. 64.71%, *p* < 0.0001). It is worth noting that two fungi (*Candida albicans and Aspergillus*) and two bacteria (*Staphylococcus aureus and Staphylococcus hominis*) were detected solely by CMT and not found in tNGS (Fig. 2C).

As shown in Figs. 2D and 92.09% (163/177) of pathogens detected by mNGS were also detected by tNGS. In terms of detection capability for Mycoplasma hominis, tNGS was inferior to mNGS (3/6 vs. 6/6, *p* = 0.046), but tNGS significantly outperformed mNGS in detecting other intracellular bacteria (11/11 vs. 7/11, *p* = 0.027). Moreover, tNGS identified a significantly higher proportion of Gram-negative bacteria (GNB) compared to mNGS (95.45% versus 86.36%, *p =* 0.036), and there is a clear difference in performance between tNGS and mNGS for fungal detection. 75% of the fungi were detected by both tNGS and mNGS, with each method detecting seven additional fungi uniquely. The additional fungal detections by tNGS were predominantly *Candida albicans* (4/7 cases), whereas the fungi exclusively identified by mNGS were more dispersed.

We further compared the RPM values of the GNB and fungi detected by only tNGS (tNGS+/mNGS-) with those detected by both tNGS and mNGS (tNGS+/mNGS+) and found a significantly lower RPM level in tNGS+/mNGS- samples (*p* < 0.05). In contrast, the RPM of the fungi and GNB exclusively detected by mNGS did not show significant differences from those commonly detected by both NGS platforms (Fig. 2E). Genomic alignment revealed that 5/7 fungi (including 3 *Candida species*, 1 *Pneumocystis jirovecii*, and 1 *Cryptococcus neoformans*) detected by mNGS had sequence fragments located at the periphery of the target regions for the corresponding pathogens in tNGS, with read overlaps of less than 35 nucleotides, leading to non-compliance with the reporting criteria (Fig. 2F). For 2/7 cases (*Trichophyton mentagrophytes* and *Mucor michiganensis*), the read fragments were within the target coverage area of tNGS but were not detected.

### The performance of ultra-broad hybrid capture-based tNGS, mNGS, and CMT in immunocompromised patients

**Fig. 3:**
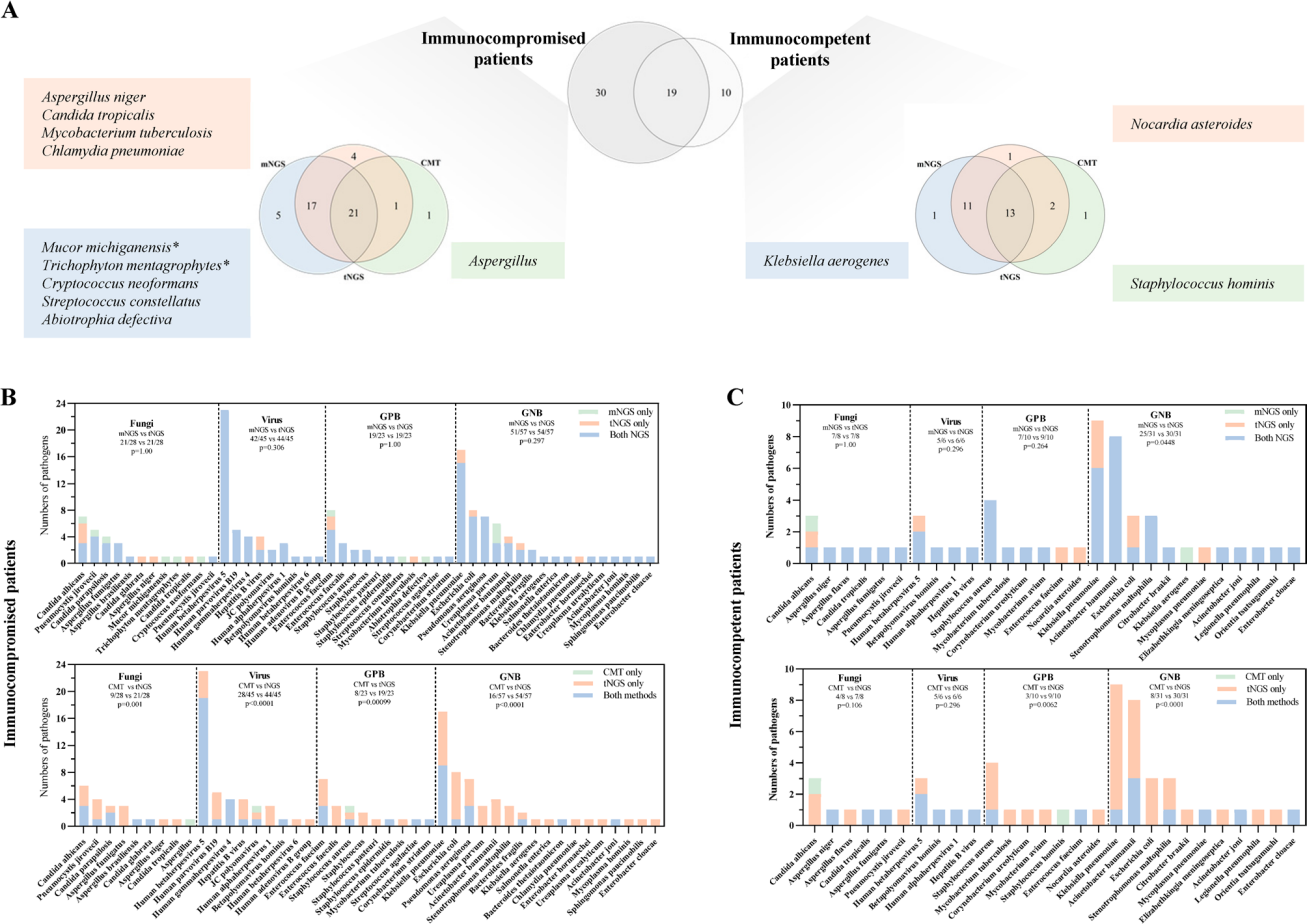
Pathogen detection performance of tNGS in immunocompromised and immunocompetent populations. (**A**) Venn plot of pathogen spectrum distribution in two populations and pathogen spectrum distribution detected by various methodologies in two groups. The “*” marks two pathogens that are not included in our tNGS detection panel. (**B**&**C**) Comparison of pathogen detection of tNGS between CMT and mNGS in immunocompromised and immunocompetent patients

The pathogen spectrum in immunocompromised populations is typically more diverse, and tNGS with limited pathogen targets may struggle to provide adequate coverage. Therefore, we specifically evaluated the coverage performance of the ultra-broad hybrid capture-based tNGS. In this study, compared to immunocompetent patients, immunocompromised patients exhibited a broader spectrum of species (49 vs. 29 kinds of species, reported by at least one method) and a clear tendency toward rarer pathogens (Fig. 3A). In the immunocompromised group, tNGS failed to detect five pathogens identified by mNGS, two of which were not included in our hybrid target panel (Supplementary Table 2). The non-targeted full-coverage feature of mNGS still holds an absolute advantage in detecting infections among immunosuppressed populations. In the immunocompetent group, all reported species were within the pathogen panel of tNGS we used, suggesting a satisfactory coverage range. In the two subgroups based on immune status, we also observed no significant difference in the detection rates of various pathogens between tNGS and mNGS, with the positive rate of tNGS being higher than that of CMT (90.20% vs. 39.87%, *p* < 0.0001) (Fig. 3B, C).

### The performance of ultra-broad hybrid capture-based tNGS, mNGS, and CMT for BSI diagnosis

**Fig. 4:**
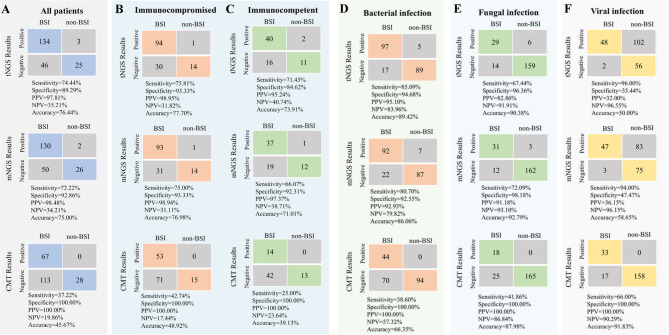
Diagnostic performance comparison of tNGS, mNGS, and CMT. (**A**, **B**, **C**) Using a predefined composite diagnostic standard as a reference, the diagnostic performance of the three methods is presented. Composite diagnostic standard: the total patients with definite, probable, and possible BSI as diagnosed BSI and unlikely BSI as non-BSI patients. (**D**–**F**) The diagnostic performance of the three methods in bacterial, fungal, and viral infections

Analysis was conducted to assess concordance with clinical diagnoses, considering patients with definite, probable, and possible BSI as diagnosed BSI and those with unlikely BSI as non-BSI patients. Based on this, tNGS identified pathogens in 134 patients with BSI, while 5 patients were determined solely by CMT and 11 solely by the mNGS method. Overall, the diagnostic accuracy of tNGS was 76.44%, which was comparable to the diagnostic accuracy of mNGS (75.00%, *p =* 0.732) but significantly higher than that of CMT (45.67%, *p* < 0.0001) (Fig. 4A). Among immunocompromised patients, the sensitivity and specificity of tNGS were 75.81% and 93.33%, respectively, with diagnostic accuracy that did not significantly differ from its performance across all patients (*p =* 0.786). The diagnostic accuracy of tNGS was comparable to that of mNGS (77.70% vs. 76.98%, *p =* 0.886), and significantly higher than that of CMT (*p* < 0.0001) (Fig. 4B). Similar findings were observed in immunocompetent individuals (Fig. 4C). For both bacterial and fungal BSI, tNGS demonstrated diagnostic accuracy similar to mNGS (*p* > 0.05). The diagnostic accuracy of tNGS for bacterial BSI was significantly higher than that of CMT. Additionally, compared with CMT, although tNGS exhibited greater sensitivity for fungal BSI (67.44% vs. 41.86%, *p* = 0.017), the diagnostic accuracy was not significantly different (90.38% vs. 87.98%, *p* = 0.430) (Fig. 4D-F). When determining BSI as those that are definite and probable BSI, the diagnostic accuracy of tNGS and mNGS remained comparable (*p =* 0.678), at 83.78% and 82.16%, respectively (Supplementary Fig. 1). The same results were consistently observed in immunocompromised individuals, with the diagnostic accuracy of tNGS and mNGS being comparable (83.87% vs. 83.06%, *p =* 0.864) and significantly higher than that of CMT (*p* < 0.0001).

### Clinical impact of tNGS testing on patient management

Based on the retrospective clinical information statistics, the impact of plasma mNGS results on clinical antibiotic treatment decisions was included to simulate the clinical impact of tNGS. 196 tNGS samples achieved similar clinical diagnostic results to those of mNGS and were used for subsequent evaluation. Based on the mNGS results, the antibiotic decision had been affected in 68.75% of patients. Among these patients, 97.20% (139/143) of tNGS results were consistent with mNGS results, which could have a positive impact in 70.92% of patients (compared with 68.75%; *p* = 0.52), including 44.90% of patients with a clear infection, 12.24% of patients with escalated or de-escalated treatment, and 13.78% of patients promoting the exclusion of infection (Table 2). Among the other 65 patients, mNGS detected microorganisms (bacteria or viruses), but did not alter the diagnosis or treatment of the patients. Among them, 84.62% (55/65) of the tNGS results matched the mNGS results, which could have a similar clinical impact. tNGS detected all two drug resistance genes reported by mNGS plus an additional 18 (Supplementary Fig. 2). However, due to the limited drug resistance information provided by both mNGS testing and CMT, this cohort was unable to evaluate the clinical impact of the additional drug resistance genes detected by tNGS. Notably, the impacts we mentioned above should be recognized as simulation-based inferences, and more observational studies are needed to validate them. In terms of clinical market prices for a single test locally (Shanghai, China), tNGS can complete the DNA and RNA workflow simultaneously for approximately $200, while mNGS testing for the DNA and RNA process costs around $900.

**Table 2:** Clinical impact of plasma mNGS and tNGS testing

Categories of clinical impact	No. (%)of mNGS Tests (*n* = 208)	No. (%)of tNGS Tests (*n* = 196)
**Positive impact**	143(68.75%)	139(70.92%)
Enabled new diagnosis of infection and initiation of targeted therapy	87(41.83%)	85(43.37%)
Enabled earlier diagnosis than conventional methods and initiation of targeted therapy	4(1.92%)	3(1.53%)
Enabled new diagnosis of infection and escalation of therapy	20(9.62%)	20(10.20%)
Enabled new diagnosis of infection and de-escalation of therapy	4(1.92%)	4(2.04%)
Enabled ruling out of Infection and initiation of noninfectious therapy	28(13.46%)	27(13.78%)
**No impact or negative impact**	65(31.25%)	55(29.08%)
Redundant information, antibiotics, and clinical plan were not changed	38(18.27%)	34(17.35%)
A negative result with no clinical significance	11(5.29%)	7(3.57%)
No relevant pathogen (considered contamination)	10(4.81%)	10(5.10%)
Patient forgoes further treatment (discharge or death)	6(2.88%)	4(3.06%)

## Discussion

The clinical management of immunocompromised patients with suspected BSI presents significant challenges due to the typically nonspecific signs and symptoms [23]. The potential clinical utility of plasma mNGS has begun to transform the field of diagnostic microbiology in patients with BSI [9, 24–27]. However, its high cost limits its potential to benefit more patients. tNGS technology can amplify signal-to-noise ratio, reduce data volume requirements, and greatly reduce costs. However, the size of pathogen panels and genome ranges targeted by tNGS methodology is limited; thus, it is currently mainly used for the detection of respiratory samples [12–15], with limited reports in plasma cfDNA detection. This study introduced an ultra-broad hybrid capture-based tNGS method, characterized by an ultra-broad pathogen panel (1872 pathogens) and high-density probe coverage. Through comparison with mNGS and CMT, as well as analysis in different subgroups, the ultra-broad hybrid capture-based tNGS has demonstrated satisfactory capability in diagnosing pathogens causing BSI.

Compared to CMT, the tNGS method has a significantly higher sample detection rate (83.65% vs. 32.21%, *p* < 0.001) and is comparable to mNGS (83.17%). 64.42% (134/208) of the tNGS results were identified as true positives. As we can see, both tNGS and mNGS would detect viruses or bacteria in the blood that are unrelated to the BSI. tNGS detected microbial nucleic acids in 37 samples, 36 of these samples were also detected by mNGS. Cell-free microbial nucleic acid fragments in plasma may originate from infected sites or non-pathogenic commensal microorganisms [11]. High-throughput sequencing technology with a broad detection range can naturally detect biological entities that are clinically insignificant (potential contaminants or non-pathogenic organisms present in the human body), especially considering the widespread presence of *herpesvirus* in the blood [28, 29]. Therefore, in most cases, clinicians still need to make empirical diagnoses and take action based on their professional knowledge, just as they do when interpreting the results of routine microbial cultures [30].

Due to the significant difference in sensitivity between current molecular diagnostic methods and traditional blood culture methods, accurately interpreting molecular detection results as positive and blood culture results as negative is challenging [9, 17, 22]. Molecular results may be due to non-viable, non-replicating bacteria, transient bacteremia/viremia, intracellular microorganisms, antibiotic effects, or contamination [31]. Therefore, we introduced multiple definitions of BSI and compared the results with mNGS [9, 17]. In our study, 34.62% (72/208) of samples were tNGS+/CMT-. Among the 18/72 samples in the definite BSI group, non-blood sample test results confirmed 15/18, and 3/18 were confirmed by clinical symptoms combined with mNGS results. In the probable BSI and possible BSI groups, 47 out of 54 samples were clarified by mNGS results and clinical presentations, and clinical manifestations supported 7 out of 54 results. In summary, we found that the diagnostic performance of tNGS in blood samples for BSI is comparable to mNGS, both in immunocompromised and immunocompetent patients.

Theoretically, the enrichment of microbial nucleic acids through probe hybridization increases the concentration, providing tNGS with better sensitivity for pathogen detection [13, 32, 33]. In this study, it has been observed that tNGS uniquely identified 9.62% (20/208) of pathogens. Additionally, tNGS detected more intracellular bacteria than mNGS (100% vs. 63.63%, *p* < 0.05), except for *Ureaplasma parvum*. This may be because intracellular bacteria are released in the bloodstream in low loads that are difficult to recognize by randomly sequenced mNGS and are more easily captured by the probes of tNGS. However, the comparable pathogen detection rates between tNGS and mNGS indicate that tNGS did not demonstrate significant sensitivity advantages, which may be attributed to certain limitations of tNGS. For instance, tNGS failed to detect *Ureaplasma parvum* in 3 out of 6 cases. Furthermore, there is a risk of missing detections due to incomplete coverage of cfDNA fragments by the probes in 7 instances where only mNGS identified fungi. Previous studies utilizing PCR-based tNGS have also revealed similar findings, indicating that the fragmentation of microbial cell-free DNA poses a risk of off-target amplification due to the use of conserved regions in PCR methods [13, 32].

While our study provides valuable insights, it is not without limitations. Firstly, as a single-center retrospective study, our findings should be validated in prospective multicenter cohorts. Secondly, although tNGS can detect a variety of viruses, it cannot accurately quantify viral load, which limits the discussion of tNGS’s performance in detecting viruses and its clinical interpretation in this study. Lastly, while the tNGS method can enrich resistance genes, the limited clinical outcome data restricts our ability to discuss its value further. More importantly, we had anticipated that the extensive pathogen panel of 1872 pathogens would cover the spectrum of pathogens detected in immunocompromised populations. However, it turned out that even in our limited cohort of 137 patients, there were still two pathogens not included in the panel. Subsequently, we plan to increase the probe coverage for fungi and certain other microorganisms that present genome coverage risks compared to mNGS data, to reduce false-negative events caused by insufficient coverage at the probe edges. mNGS continues to hold a unique advantage in terms of comprehensive coverage, while the tNGS methodology still carries the risk of missed detections. Taking all factors into consideration, with clinical costs approximately half that of mNGS and comparable diagnostic performance, tNGS highlights its potential for widespread application in the diagnosis of BSI in immunocompetent individuals. However, we must acknowledge that the clinical risks of omitting certain pathogens may outweigh the cost savings in specific situations, particularly in immunocompromised patients.

## Conclusion

In summary, our study highlights the clinical value of Ultra-broad hybrid capture-based target next-generation sequencing tNGS in detecting pathogens associated with BSI and its comparable diagnostic performance to mNGS. This underscores the potential of tNGS as an economic choice for the early BSI screen.

## Supplementary Information

Below is the link to the electronic supplementary material.

Supplementary Material 1

Supplementary Material 2

Supplementary Material 3

Supplementary Material 4

Supplementary Material 5

## Data Availability

The datasets analyzed in the current study are available at the China National Center for Bioinformation - National Genomics Data Center and can be accessed using the BioProject identifier CRA016791.

## Abbreviations

BSI: Bloodstream infections
tNGS: Targeted Next-generation sequencing
mNGS: Metagenomic next-generation sequencing
PPV: Positive predictive value
NPV: Negative predictive value
CMT: Conventional microbiological tests
RPM: Reads per million
ICU: Intensive care units
RPM: Read per million
GPB: Gram-positive bacteria
GNB: Gram-negative bacteria
FUO: Fever of unknown origin
