# Supplementary material for: Ultra-broad hybrid capture-based targeted next-generation sequencing for sensitive plasma pathogen cfDNA detection in bloodstream infections
Source: J Transl Med. 2025 Oct 31;23:1203. doi: 10.1186/s12967-025-07258-9 (PMC12576977; doi:10.1186/s12967-025-07258-9)
Supplement: Supplementary file 1 — Supplementary Material 1 [file 12967_2025_7258_MOESM1_ESM.docx]

**Supplementary Methods**

**Sample processing and Dual-nucleic acid extraction**

Nucleic acids were extracted from blood samples using a sterile body fluid pathogen DNA/RNA extraction kit (Geneplus, Suzhou, China). Nucleic acid extraction concentration required not less than 0.1 ng/μL. Blood samples were mixed with 4 volumes of lysis buffer by vortexing for 15 seconds and incubated at room temperature for 10 minutes. The mixture was then centrifuged at high speed for 1 minute. 560 μL of anhydrous ethanol was added to each centrifuge tube, mixed by vertexing for 15 seconds, and centrifuged at high speed for 1 minute. The liquid from the centrifuge tube was transferred to the adsorption column tube, and centrifuged at 6,000 g for 1 minute to discard the waste liquid. This step was repeated until all the lysate was loaded onto the adsorption column tube. Different washing buffers were added sequentially, and the mixture was centrifuged several times. The adsorption column was then transferred to a sterile, enzyme-free centrifuge tube. 35 μL of elution buffer was added to the center of the adsorption column membrane, and the mixture was incubated at room temperature for 1 minute. The mixture was centrifuged at 10,000 g for 1 minute to collect the liquid. The product could then be directly used for downstream experiments.

**Library preparation and enrichment**

cDNA synthesis and library preparation were performed with the HieffNGS®C37P4 One PotcDNA&gDNA Library Prep Kit (Yeasen, Shanghai, China) according to the protocol. An aliquot was taken through library enrichment with NadPrep® NanoBlockers (Nanodigmbio, Nanjing, China) reagents to generate the product for targeted sequencing, with the remaining saved after the post-indexing cleanup step as the product for metagenomic sequencing. Target enrichment was performed by incubating Geneplus probes(covered 1872 pathogens) with samples for approximately 4 h. The probe and bound products were captured, removed, and eluted and underwent an 18-cycle PCR for library preparation. Products for metagenomic and targeted sequencing were quantified with a Qubit 3.0 instrument using dsDNA HS reagents. Library concentration should not be less than 0.73 ng/μL. Products were stored at −20°C until Sequencing.

**Sequencing and Bioinformatic analysis**

Sequencing was performed on Gene+Seq-100 (GenePlus-Suzhou) with a 100-bp single-end read sequencing to goal depths of 5 million reads for the targeted workflow. Sequencing quality should be no less than 95% for Q20 and no less than 88% for Q30. Analysis of sequencing data generated by the workflow was a Geneplus' self-built automated Data Analysis Solution. In short, basic quality control was carried out on the sequencing data using fastp software, and host genes and ribosomal RNA were filtered after the quality control was passed. The filtered reads were compared with the self-built pathogenic microorganism database using Burrows-Wheeler Aligner software (BWA, 0.7.12-r1039), and the retained results were annotated. Resistance genes and mutations at resistance sites are annotated through BLASTn and BWA alignment against corresponding databases, with the final results compared between batches and summarized for automated machine interpretation. Microbial reads within the target range were normalized to reads per million (RPM), and only microorganisms above a predefined threshold were initial reported in this study. The threshold was set at RPM ≥6 for common pathogens (excluding mycobacteria) and ≥0.5 for fungi and mycobacteria. A manual review is conducted. Microorganisms with abnormal genomic coverage will be filtered out.
