# Supplementary figures and images for: Ultra-broad hybrid capture-based targeted next-generation sequencing for sensitive plasma pathogen cfDNA detection in bloodstream infections
Source: J Transl Med. 2025 Oct 31;23:1203. doi: 10.1186/s12967-025-07258-9 (PMC12576977; doi:10.1186/s12967-025-07258-9)

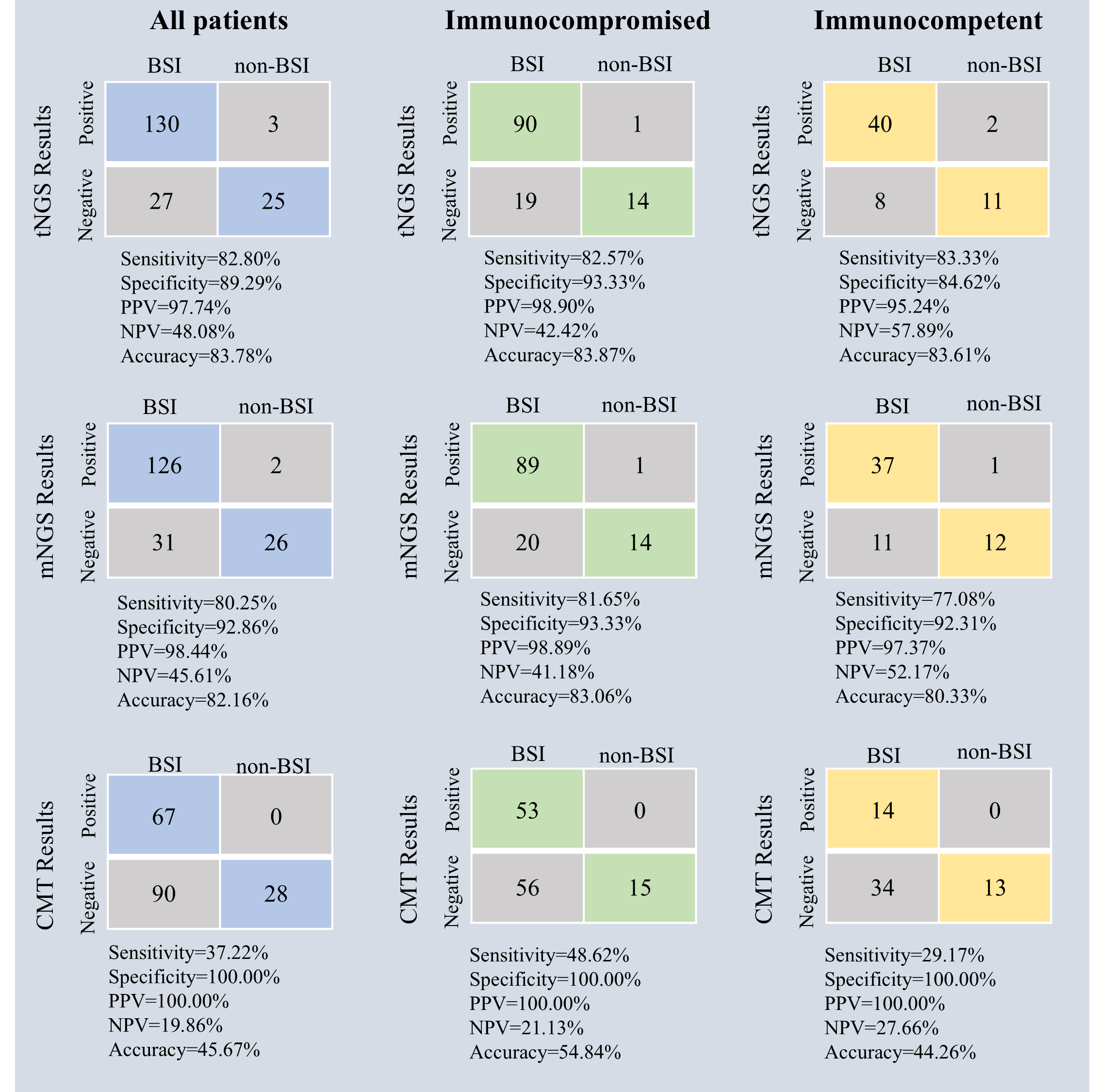

Supplement: Supplementary file 4 — Supplementary Material 4 [file 12967_2025_7258_MOESM4_ESM.tif]

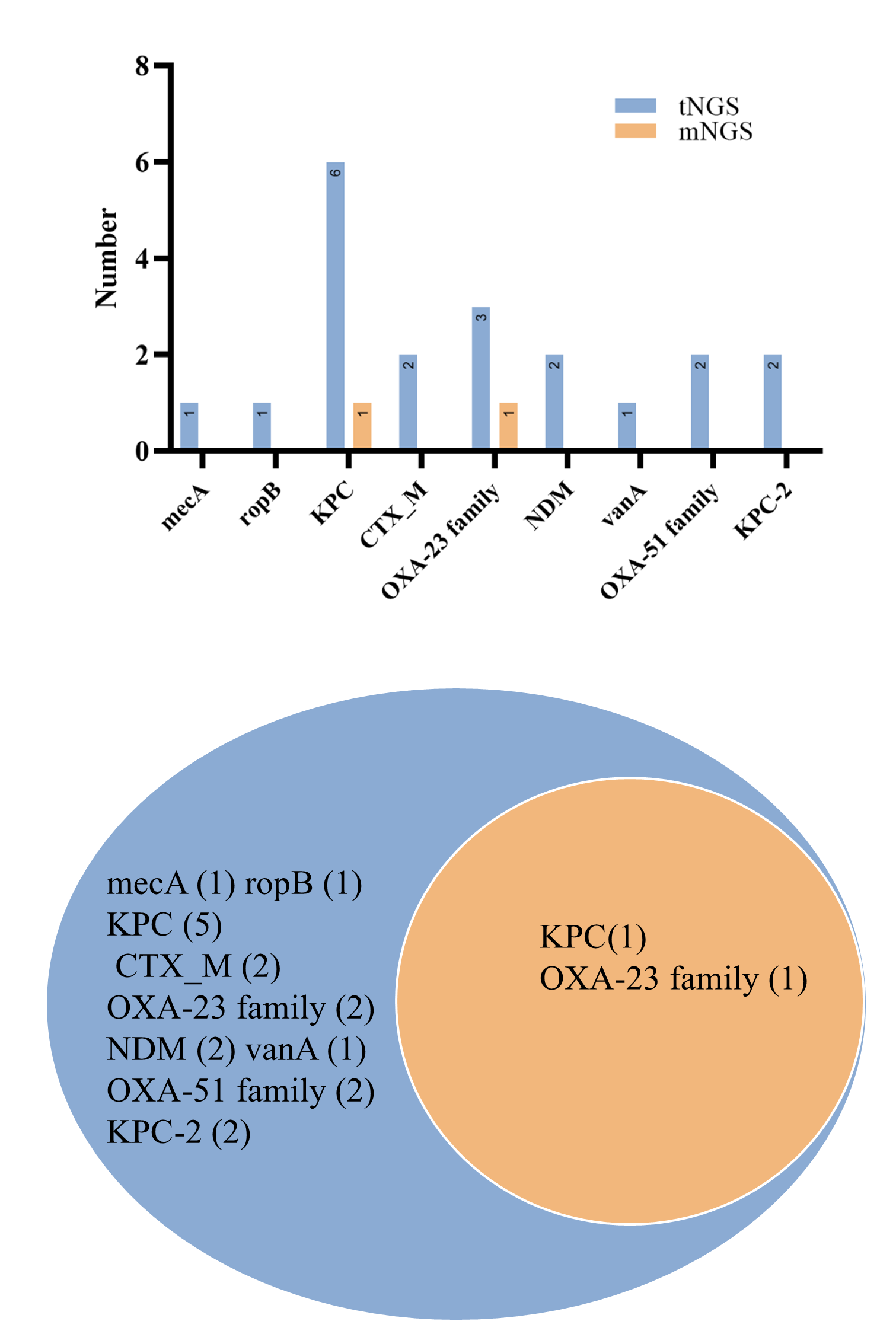

Supplement: Supplementary file 5 — Supplementary Material 5 [file 12967_2025_7258_MOESM5_ESM.tif]
